# Adipocyte Browning: A Promising Avenue in Anti-Obesity Therapy

**DOI:** 10.3390/ijms27031321

**Published:** 2026-01-28

**Authors:** Young-An Bae, Hyae Gyeong Cheon

**Affiliations:** 1 Department of Microbiology and Lee Gil Ya Cancer and Diabetes Institute, Gachon University College of Medicine, Incheon 21999, Republic of Korea; yabae03@gmail.com; 2 Department of Pharmacology and Lee Gil Ya Cancer and Diabetes Institute, Gachon University College of Medicine, Incheon 21999, Republic of Korea

**Keywords:** adipocytes, transdifferentiation, uncoupling protein 1, novel browning agents

## Abstract

Adipocyte browning refers to the inducible transdifferentiation or de novo recruitment of thermogenically active beige adipocytes within white adipose tissue depots. Beige adipocytes, characterized by multilocular lipid droplets and high mitochondrial density, express uncoupling protein 1 and possess a metabolic phenotype similar to that of classical brown adipocytes. This plasticity of adipose tissue is regulated by a complex network of transcriptional coactivators (e.g., PRDM16, PGC-1α), epigenetic modulators, non-coding RNAs, and hormonal signals. Environmental cues, such as chronic cold exposure, exercise, and caloric restriction, further potentiate browning via sympathetic nervous system activation and endocrine crosstalk. At the systemic level, adipocyte browning enhances energy expenditure, improves insulin sensitivity, and mitigates lipid accumulation, making it a promising target for the treatment of obesity, type 2 diabetes mellitus, and other metabolic syndromes. Several browning agents (natural products and repositioned drugs) and novel chemicals that induce browning have been reported. However, the translational application of these agents in humans faces challenges related to interspecies differences, depot-specific responses, and long-term safety. This review critically examines molecular regulators, existing browning agents, and the discovery of novel browning agents, with the aim of harnessing them for metabolic disease intervention.

## 1. Introduction

Obesity has emerged as a global health crisis with its prevalence reaching epidemic proportions in both developed and developing countries [1]. Obesity is characterized by the excessive accumulation of white adipose tissue (WAT) and is a major risk factor for a variety of chronic conditions, including type 2 diabetes mellitus, cardiovascular diseases, certain cancers, and metabolic syndrome [2,3]. Traditional therapeutic strategies, such as caloric restriction, increased physical activity, and pharmacological interventions, have demonstrated limited long-term success owing to challenges in adherence, efficacy, and safety [4]. Recently, GLP-1 receptor agonists, including liraglutide (Saxenda^®^) and semaglutide (Wegovy^®^), showed some effects in the reduction in body weight in the clinical setting [5,6], helping improve various metabolic parameters. However, the treatment involves risks, including gastrointestinal side effects [7], pancreatitis and gallbladder diseases, and has limitations, such as injection compliance and unequal effectiveness due to genetic factors [8]. Therefore, innovative approaches targeting different mechanistic factors are required.

One promising strategy involves enhancing energy expenditure through the activation or induction of thermogenic adipocytes [9]. Unlike WAT, which primarily functions in energy storage, brown adipose tissue (BAT) specializes in energy dissipation in the form of heat through non-shivering thermogenesis mediated by the uncoupling protein 1 (UCP1) [10]. In recent years, a distinct population of inducible thermogenic cells known as “beige” or “brite” adipocytes has garnered considerable attention [11]. These cells, which reside within WAT depots, can be activated to acquire BAT-like thermogenic features in response to specific stimuli, a process commonly referred to as adipocyte browning [12].

Adipocyte browning is a metabolic reprogramming process that transforms energy-storing white fat into energy-expanding beige fat. This phenomenon not only boosts energy expenditure but also improves systemic insulin sensitivity and lipid metabolism, thereby offering a multifaceted strategy for combating obesity and related disorders [13,14]. Emerging evidence from animal studies and early phase human trials suggests that promoting adipocyte browning could serve as a viable adjunct or alternative to current anti-obesity therapies [15,16].

This review aims to provide a comprehensive overview of the biology of adipocyte browning, detailing the underlying molecular mechanisms and signaling pathways involved, highlighting recent advances in preclinical research and emerging clinical evidence supporting adipocyte browning as a therapeutic strategy, and discussing the therapeutic potential of browning strategies in the context of anti-obesity treatment. In addition, we address the current limitations and outlines future directions for the translation of browning-based therapies into clinical practice.

The literature for this review was identified through systematic searches of PubMed, Web of Science, and Google Scholar up to August 2025. Key search terms included “adipocyte browning,” “beige adipocytes,” “brown adipose tissue,” “thermogenesis,” “UCP1,” and “anti-obesity therapy.” Priority was given to recent mechanistic in vitro and in vivo studies, human clinical trials, and high-impact review articles. When conflicting results were reported, representative studies were discussed to provide balanced perspectives.

## 2. Mechanisms of Adipocyte Browning

**Figure 1:**
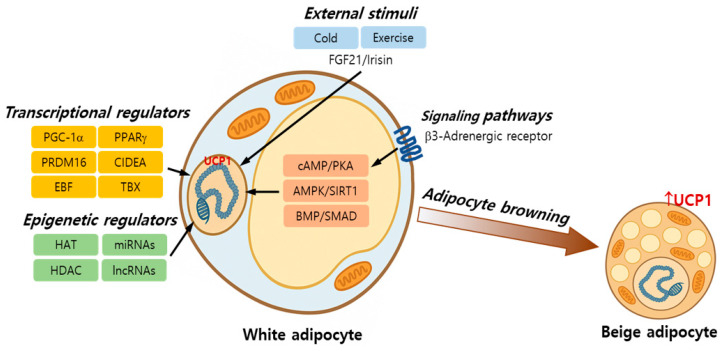
Mechanisms of adipocyte browning. Abbreviations: PGC-1α, peroxisome proliferator-activated receptor gamma coactivator-1 alpha; PPARγ, peroxisome proliferator-activated receptor gamma; PRDM16, PR domain-containing 16; CIDEA, cell death-inducing DFFA-like effector; EBF, early B cell factor 2; TBX, T-box transcription factor 1; HAT, histone acetyltransferase; HDAC, histone deacetylase; miRNA, micro RNA; lncRNA, long noncoding RNA; PKA, protein kinase A; AMPK, AMP-activated protein kinase; BMP, bone morphogenetic protein; UCP1, uncoupling protein 1.

The process of adipocyte browning involves the transdifferentiation or de novo emergence of beige adipocytes within the WAT, endowing it with thermogenic capabilities typically associated with BAT [12]. This phenomenon is driven by the complex interplay between transcriptional regulators, signaling pathways, epigenetic modulators, and environmental cues. Understanding these mechanisms is critical for developing strategies to pharmacologically or physiologically induce browning for therapeutic purposes [17]. The schematic summary of mechanisms of adipocyte browning is shown in Figure 1.

### 2.1. Transcriptional Regulators

Several key molecular players orchestrate the browning process by regulating the expression of thermogenic genes, particularly UCP1. UCP1 is a mitochondrial protein that plays a crucial role in non-shivering thermogenesis, particularly in BAT. It dissipates the proton gradient across the inner mitochondrial membrane, converting the energy from respiration into heat instead of ATP [18]. Peroxisome proliferator-activated receptor gamma coactivator-1 alpha (PGC-1α) is a master regulator of mitochondrial biogenesis and thermogenesis. It coactivates multiple transcription factors, including peroxisome proliferator-activated receptor gamma (PPARγ) and nuclear respiratory factors, to promote UCP1 expression [19]. PR domain-containing 16 (PRDM16) is a critical determinant of brown and beige adipocyte lineages. It interacts with C/EBPβ and PGC-1α to initiate and sustain the thermogenic program [20]. PPARγ, a nuclear receptor involved in adipogenesis, also contributes to browning when activated in the presence of PRDM16 and PGC-1α [21]. Other important regulators include cell death-inducing DFFA-like effector, early B cell factor 2, and T-box transcription factor 1, which further define the thermogenic identity of beige cells [11,22].

### 2.2. Signaling Pathways

Multiple extracellular signals converge to activate the intracellular pathways that trigger the browning response. Cold exposure or sympathetic nervous system activation leads to the release of norepinephrine, which binds to β3-adrenergic receptors in adipocytes. This stimulates the cyclic AMP (cAMP)/protein kinase A (PKA) pathway, resulting in enhanced lipolysis, PGC-1α activation, and UCP1 transcription [23]. AMP-activated protein kinase (AMPK) is a key energy sensor that responds to cellular energy depletion. Activation of AMPK enhances mitochondrial biogenesis and promotes browning via PGC-1α and SIRT1 activation [24]. Fibroblast growth factor 21 (FGF21) and irisin, a myokine released during exercise, also stimulate browning through PGC-1α and other downstream mediators [25,26]. Bone morphogenetic proteins (BMP)7 and 8B, the members of the TGF-β superfamily, are implicated in brown/beige adipogenesis by activating SMAD signaling and increasing UCP1 expression [27,28].

In addition to the classical cAMP–PKA pathway, cardiac natriuretic peptides represent an important parallel signaling axis regulating adipocyte browning. Atrial and B-type natriuretic peptides bind to natriuretic peptide receptor A, leading to increased intracellular cGMP levels and activation of protein kinase G. This signaling cascade promotes mitochondrial biogenesis and induces the expression of thermogenic genes, including PGC-1α and UCP1, thereby facilitating beige adipocyte differentiation [29].

### 2.3. Epigenetic and Environmental Factors

Epigenetic modifications fine-tune gene expression during browning without altering the DNA sequences [30]. Histone modifications, such as acetylation and methylation, regulate chromatin accessibility in thermogenic genes. Histone acetyltransferases (HATs) and histone deacetylases (HDACs) modulate this dynamic state, affecting browning potential [31]. Noncoding RNAs, including microRNAs and long noncoding RNAs (lncRNAs), play important roles in browning regulation [32].

External stimuli also significantly influence beige adipocyte induction. In humans, cold exposure remains the most potent physiological stimulus for adipocyte browning primarily through β3-adrenergic stimulation [33], BAT activation, with repeated mild cold acclimation increasing BAT volume, glucose uptake, and whole-body energy expenditure [34]. Exercise training can indirectly promote adipocyte browning through the release of myokines such as irisin and meteorin-like protein, although its effects on BAT activity appear more variable than those of cold exposure [26,35,36]. On the other hand, bariatric surgery has been associated with secondary increases in BAT function through hormonal and metabolic remodeling [37,38]. Compared with pharmacological approaches, these non-pharmacological strategies are generally safer but tend to exhibit smaller and more heterogeneous effect sizes.

## 3. Pharmacological Browning Agents

### 3.1. Natural Products

A broad spectrum of natural compounds has been identified as potent inducers of adipocyte browning, acting primarily through mitochondrial biogenesis, sympathetic activation, or epigenetic remodeling. Berberine, an isoquinoline alkaloid, promotes BAT mass, resulting in a significant reduction in body weight in both patients and obese mice [39]. These effects are associated with activation of the AMPK–PGC-1α axis and increased expression of UCP1 and PRDM16 in adipocytes, as well as reduced DNA methylation at the PRDM16 promoter, thereby stabilizing beige adipogenesis [40]. Curcumin, a polyphenol from *Curcuma longa*, enhances BAT activity and induces beige adipocyte formation by stimulating AMPK and mitochondrial biogenesis; in obese adolescents, curcumin supplementation improved metabolic profiles, supporting translational potential [41,42]. Resveratrol, abundant in grapes and peanuts, promotes adipose tissue browning through the SIRT1–PGC-1α pathway [43]; however, its poor bioavailability due to extensive metabolism remains a clinical challenge [44]. Green tea catechins, especially epigallocatechin gallate, activate AMPK and prolong catecholamine signaling via catechol-O-methyltransferase inhibition, thereby enhancing thermogenesis [45,46]. Quercetin induces UCP1 and SIRT1 expression, which is partly mediated by gut microbiota remodeling [47]. Capsaicin and its analogs (capsinoids) increase energy expenditure through the activation of BAT in humans [48], and act via TRPV1-mediated sympathetic activation, inducing PPARγ, PGC-1α, and UCP1 expression in WAT [49]. Additional flavonoids, such as naringenin and apigenin, induce beige adipocyte markers through PPARα signaling and ATGL/FOXO1/SIRT1-mediated lipolysis, respectively [50,51]. Other noteworthy natural molecules include celastrol (a leptin sensitizer), butyrate (a short-chain fatty acid that inhibits HDAC9 to promote browning), and β-hydroxybutyrate, which enhances UCP1 and PRDM16 via the AMPK–SIRT1–PGC-1α pathway [52,53,54].

### 3.2. Drug Repositioning

Repurposing clinically approved drugs offers a translational advantage owing to their established safety profiles. Mirabegron, a β3-adrenergic receptor agonist approved for overactive bladder, enhances BAT activity and induces WAT browning, increasing resting energy expenditure by ~150 kcal/day in humans [55]. However, high doses are associated with tachycardia and hypertension, prompting the development of localized delivery systems such as poly(lactic-co-glycolic acid) microspheres, which restrict browning effects on adipose depots while minimizing cardiovascular risks [56]. Thiazolidinediones (TZDs), potent PPARγ agonists, also induce beige adipocytes, but adverse effects such as fluid retention, cardiovascular risk, and osteoporosis limit clinical applicability [57]. Other drugs, such as metformin, indirectly promote browning by activating AMPK and enhancing FGF21 expression [58], whereas recombinant FGF21 analogs have been actively investigated for their thermogenic effects [59].

Retinoic acid is a transcriptional activator of UCP1 expression [60,61], and fenretinide, a synthetic retinoid, protects against weight gain and insulin resistance in obese mice through both retinoic acid receptor (RAR)-dependent and -independent pathways [62]. Interestingly, adapalene, a selective RARβ agonist, showed adipose browning in vitro and in vivo through RARβ-p38MAPK-ATF2 pathway [63]. Bexarotene, a retinoid X receptor agonist, also induces modest browning of the WAT in HFD-induced obese mice in a PRDM16-dependent manner [64].

Angiotensin II is an adipokine recognized for its critical role in regulating energy homeostasis [65], and stimulation of angiotensin receptor type 2 results in increased brown adipogenesis and WAT browning [66]. Losartan is a selective angiotensin receptor type 1 antagonist that induces adipocyte browning by activating apelin signaling [67].

High-throughput screening of an FDA-approved library using Ucp1-2A-GFP preadipocytes identified Sutent, an anticancer agent with receptor tyrosine kinase inhibitory activity. Oral administration of Sutent (20 mg/kg, a dose comparable to that used in clinical patients) to HFD-fed mice resulted in reduced body weight and increased thermogenesis [68].

**Figure 2:**
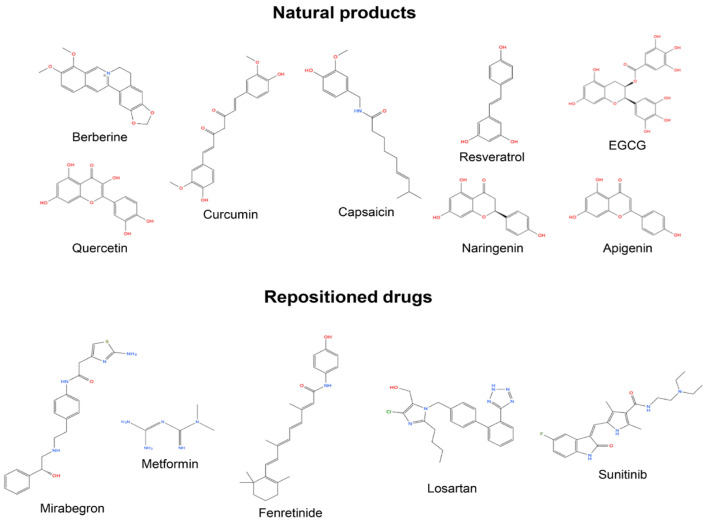
Representative chemical structures of pharmacological agents that promote adipocyte browning.

Representative chemical structures of selected natural compounds and pharmacological agents that promote adipocyte browning are shown in Figure 2. These include polyphenols (resveratrol, curcumin, EGCG), isoquinoline alkaloids (berberine), flavonoids (quercetin, naringenin, apigenin), vanilloids (capsaicin), β3-adrenergic receptor agonists (mirabegron), metabolic modulators (metformin), retinoids, AT receptor modulator (losartan), and RTK inhibitor (sunitinib).

### 3.3. Current Status of Preclinical and Clinical Studies on Browning Agents

Although numerous pharmacological agents have been reported to induce adipocyte browning, their translational status varies considerably. Most natural compounds summarized in Table 1, including berberine, resveratrol, quercetin, capsaicin/capsinoids, naringenin, apigenin, and short-chain fatty acid derivatives, have demonstrated browning activity primarily in in vitro systems and rodent models. A subset of these agents, such as green tea catechins, capsinoids, and berberine, have additionally been evaluated in small-scale human studies, where modest increases in energy expenditure or BAT activity were observed [39,45,48]. However, direct evidence for sustained white-to-beige adipocyte conversion in humans remains limited.

Among drug-repositioning candidates, mirabegron represents the most clinically advanced example. Human imaging studies using positron emission tomography–computed tomography have demonstrated β3-adrenergic receptor–mediated activation of BAT and increased resting energy expenditure [55]. Nevertheless, cardiovascular side effects, including tachycardia and elevated blood pressure at higher doses, have constrained its therapeutic window and stimulated efforts toward depot-specific delivery strategies [56]. Metformin and FGF21 analogs have progressed to clinical trials for metabolic disorders and have shown improvements in insulin sensitivity and energy metabolism, although their browning effects appear to be indirect and context-dependent [58,59].

In contrast, other pharmacological agents, including TZDs, retinoids (fenretinide, adapalene, and bexarotene), angiotensin pathway modulators, and kinase inhibitors such as sunitinib, have shown robust browning effects in preclinical models but remain limited to animal studies due to safety concerns, off-target effects, or unfavorable risk–benefit profiles [57,62,63,64,66,67,68].

Overall, while preclinical evidence strongly supports adipocyte browning as a viable anti-obesity strategy, human data remain sparse and heterogeneous. Current translational efforts increasingly emphasize tissue specificity, cardiovascular safety, and the identification of reliable biomarkers to assess beige adipocyte recruitment and thermogenic activation in humans.

## 4. Discovery of Novel Browning Agents

Advances in high-throughput screening and molecular biology have accelerated the discovery of novel small-molecule-targeting pathways central to beige adipocyte induction. Unlike classical agents such as β-adrenergic agonists, these compounds are being designed to directly target mitochondrial bioenergetics, thermogenic transcriptional regulators, or inhibitory signaling cascades, thereby inducing energy expenditure with greater selectivity and potentially fewer systemic side effects.

### 4.1. TGF-β and BMP Pathway Modulators

The TGF-β/Smad3 signaling axis suppresses beige adipogenesis, and its inhibition strongly promotes browning [69]. RepSox, a selective ALK5/TGF-β type 1 receptor inhibitor, induces robust brown adipocyte differentiation from fibroblasts, outperforming rosiglitazone in thermogenic marker induction [70]. Other TGF-β inhibitors, such as SB431542, A83-01, and LY2157299, exhibit similar browning activity [71]. Conversely, BMP7, a member of the BMP family, drives brown adipocyte differentiation and enhances UCP1 expression, whereas BMP antagonists (e.g., noggin and LDN-193189) modulate beige induction in stem cell models [27].

### 4.2. Epigenetic Regulators

Epigenetic reprogramming is a promising frontier of browning therapeutics. HDAC inhibitors such as vorinostat and entinostat increase PGC-1α and UCP1, thereby enhancing browning, promoting energy expenditure, reducing body weight, and improving glucose homeostasis in obese diabetic mice [72]. Inhibition of HDAC11 has been shown to specifically promote beige adipogenesis, independently of β-adrenergic receptor stimulation, suggesting greater selectivity with fewer systemic side effects [73]. The enhancer of zeste homolog 2 is a histone methyltransferase, and its inhibitors (e.g., GSK126) derepress thermogenic gene networks, improve cold tolerance, and reduce adiposity in preclinical studies [74]. Similarly, lysine-specific demethylase 1 inhibition facilitates BAT-specific gene expression by modulating H3K9 demethylation and activating Wnt signaling [75]. DNA methyltransferase inhibitors (e.g., azacitidine and RG108) have the potential to promote browning by upregulating thermogenic genes [76].

### 4.3. Mitochondria-Targeted Agents

Mitochondrial uncoupling is a central strategy in pharmacological thermogenesis [77]. Historically, 2,4-dinitrophenol has demonstrated potent weight-reducing effects through its protonophoric activity; however, its narrow therapeutic index and severe toxicity preclude its clinical use [78]. Recent developments in “controlled uncouplers” have revived interest in this approach. For instance, BAM15, a substituted aniline–oxadiazole protonophore, uncouples oxidative phosphorylation without elevating reactive oxygen species, increasing oxygen consumption, and improving insulin sensitivity in obese mice while avoiding hyperthermia that characterized earlier agents [79]. Using an immortalized Ucp-1 luciferase cell-based screening platform, Onodera et al. (2023) identified a novel compound [(ethyl 5-amino-4-([2-methoxyphenyl]carbamoyl)-3-methylthiophene-2-carboxylate)] that activated UCP1 and exhibited in vitro and in vivo browning activities [80].

### 4.4. Transcriptional Program Modulators

PRDM16 and PGC-1α are key regulators of thermogenic transcriptional networks. Stabilization of PRDM16 enhances beige adipocyte differentiation, while pharmacological activation of PGC-1α promotes mitochondrial biogenesis and oxidative metabolism. ZLN005, a benzothiazole derivative, enhances PGC-1α transcriptional activity, increasing mitochondrial respiration and improving glucose tolerance in preclinical models [81]. While PPARγ full agonists (TZD) promote white-to-brown adipocyte conversion but with unwanted side effects, non-TZD partial PPARγ agonist WO95E promotes browning and energy expenditure via blocking PPAR phosphorylation at S273, with minimal TZD use-associated side effects [82].

### 4.5. Notch Pathway Inhibitors

The Notch signaling cascade is a well-established suppressor of beige adipocyte differentiation. Pharmacological inhibition of γ-secretase, which prevents Notch receptor cleavage, suppresses browning. The peptidomimetic inhibitor DAPT (N-[N-(3,5-difluorophenacetyl)-L-alanyl]-S-phenylglycine t-butyl ester) induces beige adipogenesis, elevates UCP1 expression, and improves glucose tolerance in diet-induced obese mice [83]. Such compounds provide mechanistic proof that the targeted inhibition of developmental repressors can reactivate the thermogenic potential of WAT.

### 4.6. cAMP Pathway Modulators

Sympathetic stimulation via β3-adrenergic receptors induces browning primarily through cAMP–PKA signaling. Small molecules that prolong cAMP signaling have therefore gained interest as β-adrenergic mimetics. Phosphodiesterase (PDE) 3 and 4 inhibitors enhance intracellular cAMP levels, leading to sustained PKA activation and CREB-mediated transcription of thermogenic genes, thereby increasing UCP1 expression and mitochondrial respiration in adipocytes [84]. Similarly, forskolin, a diterpene activator of adenylyl cyclase, strongly induces browning responses in vitro and in vivo, especially via local delivery into subcutaneous WAT [85]. Although the proof-of-principle is strong, the systemic effects and adverse events necessitate depot-specific delivery systems to advance these candidates toward clinical application.

Beyond β-adrenergic agonists, pharmacological activation of the cGMP pathway has emerged as an alternative approach to stimulate adipocyte browning [86]. Soluble guanylyl cyclase activators increase intracellular cGMP levels and activate protein kinase G-dependent thermogenic programs [87,88], while cGMP-specific phosphodiesterase inhibitors prolong natriuretic peptide signaling [89,90]. These agents promote mitochondrial biogenesis and UCP1 expression in adipocytes, highlighting cGMP signaling as a complementary thermogenic pathway to cAMP-mediated signaling.

### 4.7. Other Emerging Probes

High-throughput screening helps to identify molecules that modulate pathways indirectly linked to adipogenesis. Inhibition of Wnt signaling using porcupine inhibitors such as IWP-2 enhances beige adipocyte differentiation from stem cells [91]. Retinoic acid derivatives, including all-trans retinoic acid and synthetic RAR agonists, also upregulate PGC-1α and UCP1, though their pleiotropic effects limit translational potential [92].

Collectively, these emerging classes of browning agents act via distinct but convergent mechanisms that enhance thermogenic programming. Compounds targeting the TGF-β/BMP axis relieve transcriptional repression and reprogram precursor cells toward a beige lineage, whereas epigenetic modulators remodel chromatin accessibility to sustain UCP1 expression. Mitochondrial uncouplers directly boost energy expenditure through controlled proton leakage, and transcriptional coactivator modulators such as PRDM16 or PGC-1α activators promote mitochondrial biogenesis and oxidative metabolism. Notch and cAMP pathway inhibitors amplify intrinsic signaling that supports beige adipocyte differentiation. Despite differences in entry points, developmental, epigenetic, and bioenergetic factors all converge on the selective induction of metabolically active beige adipocytes with minimal systemic toxicity. Preclinical examples, including BAM15, ZLN005, DAPT, and PDE inhibitors, underscore the therapeutic promise of such small molecules. Future developments should prioritize target specificity, depot-restricted delivery, and long-term safety to enable their clinical translation. The mechanisms by which these compounds promote adipocyte browning are summarized in Table 2.

## 5. Challenges and Limitations

Despite promising preclinical results, adipocyte-browning therapies face key biological and safety barriers. Inter-individual differences in BAT mass and activity, shaped by age, sex, adiposity, and genetics, lead to a wide variability in thermogenic potential [93]. Human beige fat is less abundant and responsive than rodent fat, which complicates the translation of animal data [34]. Pharmacological induction of thermogenesis, especially through β-adrenergic or mitochondrial uncoupling pathways, carries the risk of hypermetabolism, oxidative damage, and cardiovascular effects [55]. Furthermore, beige adipocytes exhibit plasticity and can revert to white adipocytes once stimulation stops, posing challenges for treatment durability [94]. Depot-specific targeting and controlled activation remain central to achieving efficacy and acceptable safety.

Recent studies have identified UCP1-independent thermogenic mechanisms that contribute to energy dissipation in beige adipocytes [95,96,97]. These mechanisms include creatine substrate cycling [95], sarcoplasmic reticulum calcium cycling mediated by SERCA2b [96], and lipid futile cycling [97]. Such pathways may partially compensate for limited UCP1 activity in human adipose tissue and offer alternative targets for therapeutic intervention [97,98]. Incorporating these mechanisms broadens the conceptual framework of adipocyte browning and underscores the need to move beyond UCP1-centric strategies.

## 6. Future Directions

To overcome the translational and regulatory barriers, future studies should focus on human-relevant models, precise biomarkers of thermogenic activation, and long-term safety evaluations. Integration of non-invasive imaging (e.g., positron emission tomography-computed tomography and magnetic resonance imaging thermometry) and multi-omics profiling will enable quantitative monitoring of browning responses. Regulatory frameworks should evolve to accommodate metabolism-targeted drugs, whose primary outcomes extend beyond weight loss to improvements in insulin sensitivity and cardiovascular function. Ultimately, combining personalized diagnostics with the targeted or depot-specific delivery of browning agents could bridge the gap between proof-of-concept and clinical implementation.

## 7. Conclusions

Adipocyte browning is a novel and biologically compelling avenue for the treatment of obesity and associated metabolic disorders. Advances in the understanding of beige adipocyte biology have revealed a highly plastic and inducible cell population capable of significantly enhancing energy expenditure and improving systemic metabolic homeostasis. Preclinical studies have demonstrated robust effects on weight regulation, insulin sensitivity, and lipid metabolism, whereas early human trials have shown promise for the pharmacologic and lifestyle-based activation of BAT.

However, translating these findings into scalable, safe, and effective therapies remains challenging. Inter-individual variability, a limited understanding of long-term outcomes, and the need for targeted delivery methods underscore the importance of continued research. As the body of evidence grows, integrated strategies that combine personalized medicine, novel pharmacological agents, and combination regimens with existing therapies may unlock the full therapeutic potential of adipocyte browning. Further clinical validation, mechanistic studies, and cross-disciplinary collaboration are essential to determine the role of browning as a viable adjunct or alternative to conventional obesity treatments.

## Figures and Tables

**Table 1:** Pharmacological agents promoting adipocyte browning.

Representative Agents	Primary Target/Pathway	Mechanism of Browning Induction	Key Metabolic Effects/ Remarks	Refs.
**Natural products**				
Berberine	AMPK → PGC-1α → UCP1, PRDM16	Activates AMPK–PGC-1α axis; demethylates PRDM16 promoter	↑ Mitochondrial biogenesis; ↓ DNA methylation; improved insulin sensitivity	[39,40]
Curcumin	AMPK activation	Stimulates mitochondrial biogenesis and UCP1 expression	↑ BAT activity; improved lipid/glucose metabolism	[41,42]
Resveratrol	SIRT1–PGC-1α	Enhances mitochondrial biogenesis via SIRT1 activation	↑ Energy expenditure; limited by low bioavailability	[43,44]
EGCG (green-tea catechin)	AMPK/COMT inhibition	Prolongs catecholamine signaling; enhances thermogenesis	↑ Energy expenditure in humans	[45,46]
Quercetin	SIRT1	Up-regulates UCP1 and SIRT1; modulates gut microbiota	↓ Inflammation; ↑ thermogenesis	[47]
Capsaicin/Capsinoids	TRPV1	Sympathetic activation via TRPV1	↑ UCP1 and PPARγ; ↑ energy expenditure	[48,49]
Naringenin/Apigenin	PPARα/ATGL–FOXO1–SIRT1	Induces lipolysis-linked browning	↓ Obesity, ↑ lipid oxidation	[50,51]
Celastrol/Butyrate/ β-Hydroxy butyrate	AMPK–SIRT1–PGC-1α	Leptin sensitization/HDAC9 inhibition/ ketone signaling	↑ Thermogenic gene expression; anti-obesity effects	[52,53,54]
**Drug repositioning**				
Mirabegron	β3-Adrenergic receptor	Activates cAMP–PKA–UCP1 axis	↑ BAT activity, ↑ energy expenditure (~150 kcal/day in humans)	[55,56]
TZDs	PPARγ	Induce beige adipocyte differentiation	↑ Browning; limited by side-effects (edema, bone loss)	[57]
Metformin/FGF21 analogs	AMPK → FGF21	Indirect activation of thermogenesis	↑ Mitochondrial biogenesis, ↑ glucose tolerance	[58,59]
Retinoids (fenretinide, adapalene, bexarotene)	RAR/RXR	Up-regulate UCP1 via RAR–p38MAPK–ATF2 pathway	Browning in vivo; improved insulin sensitivity	[60,61,62,63,64]
Losartan/Ang II pathway	AT1R/AT2R– apelin	AT1R blockade or AT2R stimulation promotes browning	↑ PRDM16, ↑ UCP1; reduced obesity in mice	[65,66,67]
Sutent (sunitinib)	RTK inhibitor	Directly increases UCP1 expression	↓ Body weight; ↑ thermogenesis in HFD mice	[68]

Summary of pharmacological compounds known to induce white-to-beige adipocyte conversion via diverse signaling pathways. Mechanistic diversity spans AMPK activation, β-adrenergic stimulation, transcriptional, and epigenetic modulation. Abbreviations: AMPK, AMP-activated protein kinase; PGC-1α, proliferator-activated receptor gamma coactivator-1 alpha; UCP1, uncoupling protein 1; PRDM16, PR domain-containing 16; COMT, catechol O-methyltransferase; TRPV1, transient receptor potential vanilloid 1; PPAR, peroxisome proliferator-activated receptor; ATGL, adipose triglyceride lipase; FOXO, forkhead box O; FGF21, fibroblast growth factor 21; RAR, retinoic acid receptor; RXR, retinoid X receptor; AT1R, angiotensin receptor type 1; AT2R, angiotensin receptor type 2; RTK, receptor tyrosine kinase; EGCG, epigallocatechin gallate; TZD, thiazolidinedione; ↑, increase; ↓, decrease.

**Table 2:** Emerging small molecules that promote adipocyte browning.

Cmpd	Chemical Information	Primary Target	Mechanism	Reported Biological Effects	Refs.
RepSox	2-[5-(6-methylpyridin-2-yl)-1H-pyrazol-4-yl]-1,5-naphthyridine	ALK5/TGFβ R1 inhibition	Reprogramming by inhibiting the pathway and inducing the transcription factor Nanog	Reprogram iPSC by inhibiting TGFβ signaling	[70,71]
Vorinostat	N-hydroxy-N’-phenyloctanediamide	HDAC inhibition	Increase PGC-1α and UCP1	Improve glucose homeostasis	[72]
Entinostat	(Pyridin-3-yl)methyl 4-(2-aminophenylcarbamoyl)benzylcarbamate	HDAC inhibition	Increase PGC-1α and UCP1	Improve glucose homeostasis	[72]
GSK126	1-[(2*S*)-butan-2-yl]-N-[(4,6-dimethyl-2-oxo-1H-pyridin-3-yl)methyl]-3-methyl-6-(6-piperazin-1-yl-3-pyridinyl)indole-4-carboxamide	EZH2 inhibition	Inhibit histone methyltransferase to improve thermogenic gene networks	Improve cold tolerance, reduce adiposity	[74]
GSK690	N-(2-phenyl-cyclopropyl)-4- piperidinamine	LSD1 demethylase inhibition	Inhibition of oxidative phosphorylation	Reduce weight gain when fed a high-fat diet	[75]
Azacitidine	4-amino-1-β-D-ribofuranosyl-s-triazin-2(1H)-one	DNMT inhibition	Upregulate thermogenic genes	Promote adipose browning	[76]
BAM15	N^5^,N^6^-bis(2-fluorophenyl)[1,2,5]oxadiazolo[3,4-b]pyrazine-5,6-diamine	Mitochondrial proton gradient (uncoupler)	Increases proton leak across mitochondrial membrane	Enhances energy expenditure, reduces fat mass, improves insulin sensitivity in mice without hyperthermia	[79]
ZLN005	2-(4-tert-butylphenyl)-1H-benzimidazole	PGC-1α activation	Enhances PGC-1α transcriptional activity/increases mitochondrial respiration	Improve glucose tolerance	[81]
WO95E	4′-((5-((3-hydroxybenzyl)carbamoyl)-2,3-dimethyl-1H-indol-1-yl)methyl)-[1,1′-biphenyl]-2-carboxylic acid	PPARγ partial agonism	Block PPAR serine 273 phosphorylation	Increase energy expenditure	[82]
DAPT	N-[N-(3,5-difluorophenacetyl)-L-alanyl]-S-phenylglycine t-butyl ester	γ-Secretase/Notch signaling	Blocks Notch, derepresses PPARγ, and thermogenic gene expression	Promotes beige adipogenesis, improves insulin sensitivity, reduces hepatic steatosis	[83]
Rolipram	4-(3-cyclopentyloxy-4-methoxyphenyl)pyrrolidin-2-one	PDE4 inhibition	Inhibits PDE4 → ↑cAMP–PKA signaling	Induces UCP1, browning of WAT, improves metabolic health in obese mice	[84]
Cilostazol	6-[4-(1-cyclohexyl-1H-tetrazol-5-yl)butoxy]-3,4- dihydro-2(1H)-quinolinone	PDE3B inhibition	Enhances β-adrenergic cAMP signaling	Potentiates thermogenesis, increases lipolysis	[84]
Forskolin	(3R,4aR,5S,6S,8aS)-5-(acetyloxy)-3-ethenyl-4a,7-dihydroxy-8,8-dimethyl-1,6,7,8atetrahydrocyclopenta[c]chromen-9-one	Adenylyl cyclase activation	Direct cAMP elevation, PKA activation	Promotes beige adipogenesis, increases UCP1	[85]
Praliciguat	(1R,2S,4S)-4-{[(4,5-dihydro-1H-imidazol-2-yl)amino]methyl}-2-(6-fluoro-1H-indol-3-yl)cyclopropane-1-carboxamide	Guanylyl cyclase activation	Direct cGMP elevation, PI3K activation	Increase energy utilization	[88]
Sildenafil	1-[[3-(6,7-dihydro-1-methyl-7-oxo-3-propyl-1H-pyrazolo[4,3-d]pyrimidin-5-yl)-4-ethoxyphenyl]sulfonyl]-4-methylpiperazine	PDE5 inhibition	Direct cGMP elevation Catecholamine increase	Induces browning of WAT in human adults	[90]
IWP2	N-(6-methyl-2-benzothiazolyl)-2-[(3,4,6,7-tetrahydro-4-oxo-3-phenylthieno [2-d]pyrimidin-2-yl)thio]-acetamide N-(6-methyl-2-benzothiazolyl)-2-[(3,4,6,7-tetrahydro-4-oxo-3-phenylthieno [2-d]pyrimidin-2-yl)thio]-acetamide	Wnt inhibitor	Target adipocyte precursors-Enhance thermogenic properties	Induce epididymal adipocyte browning	[91]

Abbreviations: ALK, activin-like receptor kinase; EZH2, enhancer of zeste homolog 2; LSD1, lysine-specific demethylase 1; DNMT, DNA methyltransferase; PDE, phosphodiesterase; PKA, protein kinase A.

## Data Availability

No new data were created or analyzed in this study. Data sharing is not applicable to this article.
